# Simultaneous multiplexed quantification of caffeine and its major metabolites theobromine and paraxanthine using surface-enhanced Raman scattering

**DOI:** 10.1007/s00216-015-9004-8

**Published:** 2015-09-07

**Authors:** Omar Alharbi, Yun Xu, Royston Goodacre

**Affiliations:** School of Chemistry, Manchester Institute of Biotechnology, University of Manchester, 131 Princess Street, Manchester, M1 7DN UK

**Keywords:** Nanoparticles, Nanotechnology, Spectroscopy, Instrumentation, IR spectroscopy, Raman spectroscopy, Forensics, Toxicology, SERS

## Abstract

Accurate quantitative measurement of drugs and their metabolites is important as this can be used to establish long-term abuse of illicit materials as well as establish accurate drug dosing for legal therapeutics. However, the levels of drugs and xenometabolites found in human body fluids necessitate methods that are highly sensitive as well as reproducible with the potential for portability. Raman spectroscopy does offer excellent reproducibility, portability and chemical specificity, but unfortunately, the Raman effect is generally too weak unless it is enhanced. We therefore developed surface-enhanced Raman scattering (SERS) and combined it with the powerful machine learning technique of artificial neural networks to enable rapid quantification of caffeine and its two major metabolites theobromine and paraxanthine. We established a three-way mixture analysis from 10^−5^ to 10^−7^ mol/dm^3^, and excellent predictions were generated for all three analytes in tertiary mixtures. The range we selected reflects the levels found in human body fluids, and the typical errors for our portable SERS analysis were 1.7 × 10^−6^ mol/dm^3^ for caffeine, 8.8 × 10^−7^ mol/dm^3^ for theobromine and 9.6 × 10^−7^ mol/dm^3^ for paraxanthine. We believe this demonstrates the exciting prospect of using SERS for the quantitative analysis of multiple analytes simultaneously without recourse to lengthy and time-consuming chromatography, a method that often has to be combined with mass spectrometry.

**Electronic supplementary material:**

The online version of this article (doi:10.1007/s00216-015-9004-8) contains supplementary material, which is available to authorized users.

## Introduction

Raman spectroscopy has been proven to be a powerful physiochemical technique that can provide highly specific molecular information about a sample under analysis and allows unambiguous analyte structural characterisation [1]. This is essential in the case of detection, identification and quantification of legal drugs and illicit substances, and Raman spectroscopy has therefore been used extensively for the identification of drugs of abuse [2–5].

Although Raman spectroscopy is a useful technique that can be deployed in a portable manner, a major limitation of its use is the fact that the signal from the normal Raman process is unfortunately inherently weak. Significant enhancement of the signal can be accomplished using either resonance Raman or surface-enhanced Raman scattering (SERS). When using SERS, it is necessary for the analyte to be either in direct contact or close to a roughed metal surface [6–10], and for quantitative analysis, colloidal preparations of silver or gold nanoparticles are usually employed [11, 12]. In our laboratory, initial work has been carried out for optimising SERS for trace detection of human drugs [13] as well as illicit materials and legal highs [14, 15].

Many studies in this field have focused on the detection of a single analyte, and therefore, illicit substance detection has been limited to the detection of the specific xenobiotic. Due to the pharmacodynamics of drug metabolism, which usually occurs in the liver, if one could measure the major drug metabolites, this would allow the establishment of long-term abuse of illicit materials. This of course has the major advantage that if the drug itself is not present in any human body fluids, after it has been modified in the liver, its xenometabolites might still be present.

Chromatography linked with mass spectrometry has been used for detection, identification and quantification of drugs and their metabolites [16]. However, this requires specialist expensive equipment, is labour intensive as well as time consuming and is not generally considered field portable. By contrast, it has recently been shown that SERS offers considerable potential for drug detection and quantification and that this approach can readily be coupled with Raman spectrometry [13, 15], and we have presented recent evidence that SERS is powerful when combined with chemometrics to be used for the quantitative analysis of the drug nicotine and its major metabolites cotinine and *trans*-3-hydroxycotinine [17]. We have also developed SERS for the quantitative detection of DNA sequences from three bacterial pathogens [18].

In this study, we aimed to investigate the drug caffeine, which occurs in many foodstuffs (including tea, coffee and chocolate), and its major xenometabolites. Caffeine breaks down in the liver within 3–5 h after consumption where it is converted to the metabolites paraxanthine (80 %), theobromine (12 %) and theophylline (7 %) [19]. Caffeine is readily measured in human serum and, in epidemiological studies, is one of the most variable small molecules measured [20], and this reflects its variable (person-specific) consumption levels.

**Fig. 1:**
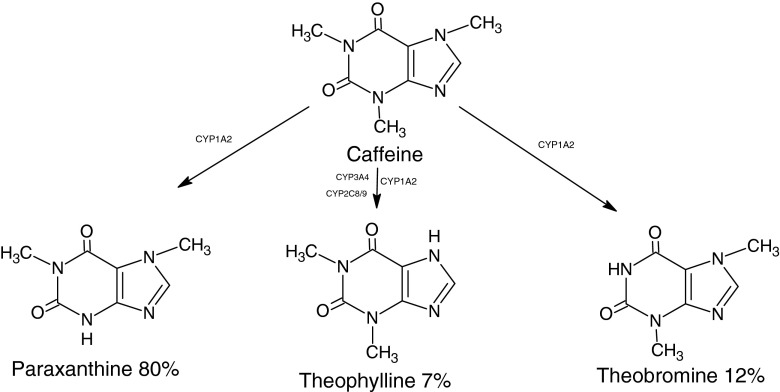
Caffeine and its immediate metabolites; *CYP* refers to the enzymes catalysing the reactions

As shown in Fig. 1, caffeine is metabolised into the major metabolites paraxanthine (80 %) and theobromine (12 %). Therefore, it is reasonable to assume that these metabolic products can be detected in human body fluids, whilst theophylline being only a modest 7 % is likely to be rather difficult to detect [21]. Based on this assumption, in this study, the drug caffeine and its two major metabolites (theobromine and paraxanthine) were prepared in tertiary mixtures to establish if SERS can be deployed for the detection and quantification of multiple analytes simultaneously, without resorting to the more complicated and time-consuming chromatographic separation and mass spectrometry techniques mentioned earlier.

## Experimental

### Chemicals

The reagents used in this investigation were silver nitrate (>99 %), trisodium citrate, potassium nitrate, sodium borohydride (NaBH_4_) (98 + %), caffeine (≥99 %), theobromine (∼98 %) and paraxanthine (∼98 %), all of which were purchased from Sigma-Aldrich (Dorset, UK) and supplied as racemates. Acetic acid (analytical reagent grade) was purchased from Fisher Scientific Company (Loughborough, UK), and sodium hydroxide standard solution (0.1 mol/L) was obtained from Riedel de-Haen Company (Seelze, Germany).

### SERS colloid preparation

#### Silver citrate colloid

Silver citrate colloid was prepared using the Lee and Meisel method [22]. Briefly, 90 mg of silver nitrate was dissolved in 500 mL of deionised water, and after which, the solution was heated to its boiling point. Ten millilitres of 1 % trisodium citrate was then added to the boiling silver nitrate solution drop by drop whilst the solution was vigorously stirred. The mixed solution was kept boiling for a further 10 min. A green-grey silver colloid was obtained, which proved to be stable at room temperature in glass conical flask covered with foil for several weeks.

#### Silver borohydride colloid

Ag nanoparticles were prepared by the reduction of AgNO_3_ using NaBH_4_ aqueous solution as a reducing agent, following the method of Lee and Meisel [22]. AgNO_3_ (10^−3^ mol/dm^3^, 25 mL) was added to an ice-cold solution of vigorously stirred NaBH_4_ (2 × 10^−3^ mol/dm^3^, 75 mL) to form a yellow colloid of Ag nanoparticles. This colloid was also stable when stored in the dark at room temperature.

#### Gold citrate colloid

Gold nanoparticles were synthesised according to a protocol described by Turkevich and colleagues [23]. In brief, 100 mL of HAuCl_4_ solution (containing 50 mg) was added to 850 mL of boiling water under vigorous stirring. Once the solution had returned to a boiling, 50 mL of 1 % trisodium citrate was added. After 30 min of continuous boiling and stirring, the gold nanoparticle solution was left to cool at room temperature. This colloid was also stable when stored in the dark at room temperature.

#### Colloid characterisation

Multiple batches of these three colloids were assessed by UV-visible spectroscopy (Thermo BioMate 5; Thermo Fisher Scientific, Inc., MA, USA), and spectra were very similar to those we had synthesised previously [15, 17, 24]. Initial SERS optimisation established that the silver borohydride colloid and gold citrate colloid did not produce very reproducible SERS data and thus were abandoned in this investigation.

Electron microscopy of multiple batches of the silver citrate colloid using a Zeiss Supra 40 VP field-emission gun scanning electron microscope (FEG-SEM; Carl Zeiss SMT GmBH, Oberkochen, Germany) operating at a voltage of 1 kV established that we had produced nanoparticles of 70 ± 30 nm (data not shown).

### Raman spectroscopy

As detailed in Alharbi et al. [17], Raman spectra were obtained using a DeltaNu® Advantage 200A (DeltaNu, Inc., Laramie, Wyoming, WY, USA) portable Raman probe. This probe was equipped with a 633-nm HeNe laser providing approximately 3 mW of power on the sample. The spectral range used was 200 to 3400 cm^−1^ with a spectral resolution of 8 cm^−1^. All spectra acquired in this study were from 30 s exposure of the analyte(s)-colloid preparations.

As in our usual practice [17], daily calibration of the instrument was achieved by obtaining the Raman spectrum of polystyrene using the calibration routine built into the instrument manufacture’s software. The spectrometer was controlled using DeltaNu, NuSpec™ software.

### SERS analyses

For SERS measurements, the following protocol was used: 200 μL of either caffeine, theobromine or paraxanthine was added to 200 μL of the colloid, and the mixture was left for 10 min to equilibrate before the addition of the aggregating agent (50 μL of 1 mol/dm^3^ NaCl). Directly after that, these mixtures were placed into the Raman system, where spectra were collected using a 633-nm laser and each reading took 30 s.

Where necessary before aggregation, the mixtures were adjusted to the required pH value by the addition of aqueous solutions of 1 mol/dm^3^ acetic acid or 1 mol/dm^3^ sodium hydroxide. One sample of each pH was prepared, five reading were recorded, and the averages were used in plots of intensity versus Raman shift recorded for a pH range of 2–12 for the analytes investigated.

### Artificial neural network analysis

Prior to any chemometric analysis, the SERS data were baseline corrected using an asymmetric least squares (ALS) algorithm and, after which, standard normal variate (SNV) normalisation was applied as detailed in Alharbi et al. [17]. This and subsequent principal component analysis (PCA) and artificial neural network (ANN) analyses were carried out in Matlab version R2014a (The MathWorks, Natick, MA, USA) as detailed in Alharbi et al. [17].

ANNs are powerful supervised learning methods, and we used these to predict the concentration levels of caffeine, theobromine and paraxanthine in tertiary mixtures. The output from SERS spectra consisted of 1024 wavenumber shifts (from 200 to 3400 cm^−1^; equally spaced), and these were fed into the input nodes of the ANNs. We used three layers in our ANNs. In addition to the 1024 inputs, we have previously established that 20 nodes in the hidden layer are appropriate for Raman spectra with this many inputs [17] and so we used 20 nodes in the hidden layer. As also tested in Alharbi et al. [17], the output layer consisted of (i) a single node, which model predicted one analyte only and so three 1024-20-1 ANNs were constructed, and (ii) alternatively, three nodes were used (one for each analyte) and this model predicted the three analytes simultaneously. All ANNs were calibrated using the scaled conjugate gradient algorithm, and the ANN models typically converged after 20–100 epochs; an epoch is when the total data used to calibrate/train the ANN were presented before the weights between the hidden layers were updated.

Validation of supervised methods is vital to test model stability, and we have employed the following procedure before [17]. Therefore, in order to test the validity of the ANN models, a bootstrapping random resampling procedure was employed. In each bootstrapping iteration, *n* samples (*n* = total number of samples) were randomly selected with replacement (i.e. one particular sample could be selected multiple times) and used as the training set. The samples that had not been selected at all were used as the *test set*. Within the training sets, ∼70 % of samples were used for training (i.e. the inner training set) and the remaining ∼30 % of samples from the training data were used as the validation/tuning set. The regression model was built on the inner training set. The model parameters that yielded the best prediction accuracy of the validation set were chosen for the final regression model, and this model was then applied to the *test sets* from the bootstrap selections. A total number of 1000 bootstrapping iterations were performed, and the prediction accuracies of these *test set iterations* were then averaged and reported. The prediction accuracies were presented in terms of validated correlation coefficient (*Q*^2^) and root mean square error of prediction (RMSEP) as in Eqs. 1 and 2.

1 $$ {Q}^2=1-\frac{{\displaystyle {\sum}_{i=1}^n{\left({\widehat{y}}_i-{y}_i\right)}^2}}{{\displaystyle {\sum}_{i=1}^n{\left({y}_{\mathrm{i}}-\overline{y}\right)}^2}} $$

2 $$ \mathrm{RMSEP}=\sqrt{\frac{{\displaystyle {\sum}_{i=1}^n{\left({\widehat{y}}_i-{y}_i\right)}^2}}{n}} $$

Where *ŷ*_*i*_ is the predicted relative concentration of sample *i*, *y*_*i*_ is the actual relative concentration of sample *i*, $\overline{y}$ is the averaged relative concentration of all the samples in the test set, and *n* is the total number of samples in the test.

## Results and discussion

### SERS optimisation experiments

SERS is a complex process that necessitates that (i) the analyte under investigation associates with the metal surfaces of the nanoparticle suspension, (ii) appropriate aggregation occurs using either salts and/or controlling pH conditions, and (iii) that this process is reproducible. Thus, rigorous optimisation of SERS is necessary. Whilst we have explored SERS optimisation for the analysis of individual analytes [12, 13, 15], when multiple analytes are to be investigated, it is important that one analyte does not dominate otherwise the others may not be seen, and the SERS signal can be highly variable [25]. Therefore, we systematically optimised the analyte-sol mixture for each of the analytes (caffeine, theobromine and paraxanthine) individually before we conducted our tertiary mixture analysis.

Below, we briefly report the optimisation of the appropriate colloid, the best aggregating agent and appropriate pH conditions, the aggregation time and also the analyte-sol association time. Whilst below these appear to be detailed in a specific order, in reality, this optimisation process occurred simultaneously and iteratively.

As described earlier (Section 3.2), we prepared and characterised (by UV and EM) silver citrate colloids, silver borohydride colloids and gold citrate colloids. Initial experiments conducted with the three analytes established that silver borohydride colloids and gold citrate colloids were not very reproducible; in addition, for silver borohydride colloids, the SERS spectra were dominated by theobromine and, for gold citrate colloids, by paraxanthine (data not shown). Therefore, we only concentrate here on the optimisation process for the silver citrate colloids. Three separate batches of silver citrate colloids were prepared to assess batch-to-batch reproducibility and to select the most reproducible sol.

Each of the three silver citrate colloid batches prepared were assessed by UV-visible spectroscopy, and batches 2 and 3 showed peak maxima at ca. 430 nm, which indicates the presence of silver [26]. By contrast, batch 1 had a broader peak extending from around 420 to 520 nm, indicating a wider particle size distribution, and this helped explain why this batch was not so reproducible.

As potassium nitrate and sodium chloride are popular aggregating agents for silver colloids [27], we prepared aqueous solutions of 0.5 and 1.0 mol/dm^3^ and these were used for optimising the aggregating agent with the three analytes. By comparing repeat aggregations (*n* = 5) of the three analytes and the SERS spectral reproducibility, we established that 1.0 mol/dm^3^ NaCl produced the most reproducible results and therefore was selected as the most appropriate aggregating agent.

**Fig. 2:**
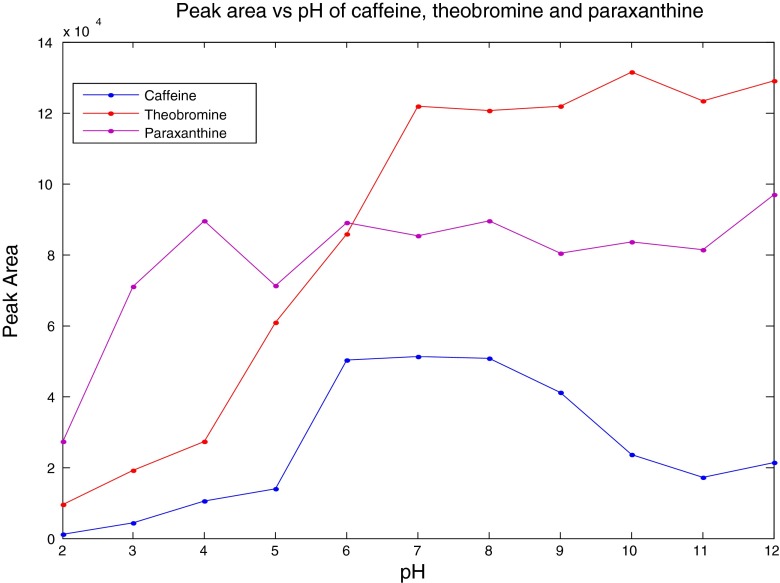
Plots of SERS peak area for the C=O deformation band at 693 cm^−1^ against pH of the SERS association for caffeine and paraxanthine, whilst the peak at 1303 cm^−1^ (*ν*(C–N) + *ρ*(CH_3_)) is plotted for theobromine. Points are the average of five repeat measurements, and the % relative standard deviations were between 0.8 and 2.7 %

Next, we optimised the pH of the analyte-sol suspension as this is known to affect the propensity for an analyte to associate with the surface. This is due to the fact that our silver colloid has citrate on the surface and is thus negatively charged, and for some analytes, the optimisation of pH is important to either neutralise the charge or to protonate the analyte so that it associates with the silver surface [17, 28]. This pH profiling for each of the analytes is shown in Fig. 2 (which is a plot of pH against peak areas for each of the analytes), and the raw spectra are reported in Electronic Supplementary Material (ESM) Figs. S1, S2 and S3. For each pH value, the peak areas of five repeats were averaged and used in the plots. The peak areas were calculated using trapezoidal numerical integration [29] on the baseline-corrected characteristic peak for each analyte (see below). Each of the analytes has different profiles: caffeine has an enhanced signal between pH 6 and 8, paraxanthine needs to be above pH 3 for maximal signal and theobromine above pH 7. As a suitable compromise, we therefore selected pH 8 as the optimal pH for all further experimentation.

The differences in these responses as a function of pH and the resultant changes in the SERS profiles for each of the analytes are clearly different (ESM Figs. S1, S2 and S3). The *p*K_a_ of the molecule is a good indication of its ionisation state under different pH conditions: caffeine has a *p*K_a_ of 10.4, theobromine’s pK_a_ is 9.9, and paraxanthine has a *p*K_a_ of 10.8 (Drug Bank, http://www.drugbank.ca; Human Metabolome Database, http://www.hmdb.ca). The N–H group in the pyrimidine ring of theobromine and paraxanthine can be deprotonated (–N^−^–) under alkaline conditions, whilst under acidic conditions, the =N–N can be protonated to =NH^+^– in the imidazole ring (and full chemical descriptions of the molecules are available in http://www.chemicalize.org). This may go some way to explain the SERS spectra differences that we observe as this change in ionisation in specific places on each of the molecules will result in each of the molecules interacting differently with the negatively charged surface of the silver nanoparticles. Of course, exactly how that interaction occurs is currently very different to predict, as computational approaches, whilst being very good at predicting Raman spectra are not yet perfected for including metal particles which are needed to predict SERS spectra.

It is also known that the time for the aggregation to occur is vital in order to get reproducible SERS. If the aggregation is dynamic, then quantification is difficult as one must collect data within a very narrow time window. We therefore collected data immediately after adding the aggregating agent for 30 min, collecting spectra every 30 s. Rather than using visible inspection of the spectra by eye, we used principal component analysis (PCA) of the full spectral data set (180 spectra) and plotted the first two PC scores as these explained the most variance in these data. For each of the three batches of silver citrate colloid, we constructed PCA score biplots (ESM Fig. S4) and it is clear that in batches 1 and 2 there is no effect on the spectra with respect to time; by contrast, batch 3 shows spectral changes as a function of time, again highlighting why it was not so reproducible. This may be because of the wider particle distribution (*vide supra*); hence, based on UV-visible spectroscopy and the PCA score biplots, batches 1 and 3 were discarded and batch 2 was selected for further optimisation. From the PCA plot of batch 2 (ESM Fig. S4B), it is clear that all spectra are identical irrespective of the aggregation time and, therefore, we chose to take measurements immediately after the aggregating agent was added.

**Fig. 3:**
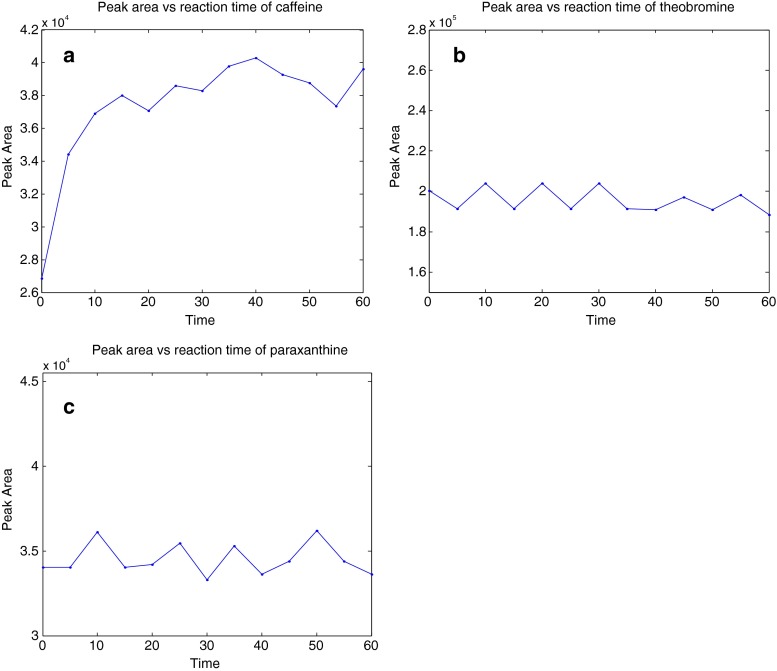
Plots of the association time for caffeine (**a**), theobromine (**b**) and paraxanthine (**c**) using 693 cm^−1^ for caffeine and paraxanthine and 1303 cm^−1^ for theobromine

Finally, the time at which the analyte was allowed to associate with the colloid was optimised. Whilst this obviously is conducted first in the actual order of performing a SERS measurement, we report it here as the above process was of course performed iteratively to establish the most optimal SERS conditions. The analyte and sol were allowed to associate for up to 60 min prior to aggregation with spectra collected every 5 min. Plots of time versus the peak areas for each of the three analytes (Fig. 3) established that theobromine and paraxanthine associated almost instantaneously with the silver surface, whilst there was slower association for caffeine, which started to plateau after 10 min. We therefore allowed the analytes in the tertiary mixture 10 min to associate with the sol prior to aggregation.

**Fig. 4:**
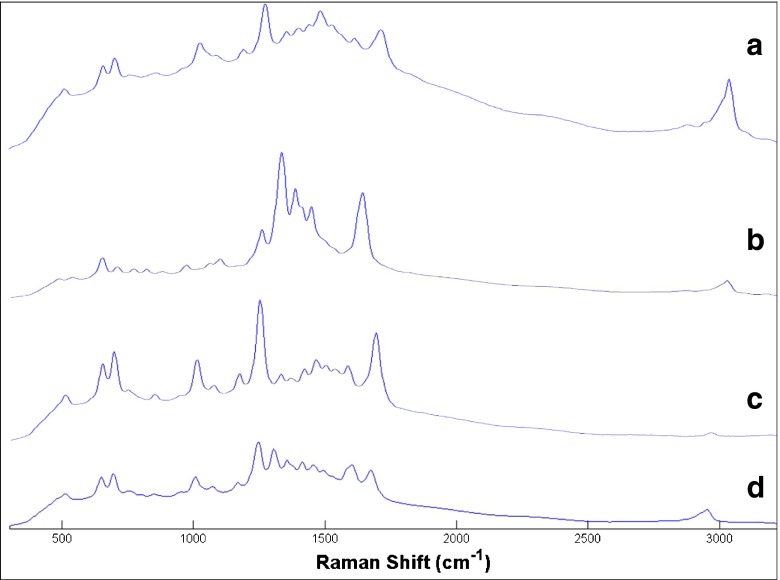
Raw SERS spectra of caffeine (**a**), theobromine (**b**) and paraxanthine (**c**) (all at 0.01 mol/dm^3^) and a 50:40:10 ratio of caffeine/theobromine/paraxanthine mixture (**d**). A silver citrate colloid and a 1 mol/dm^3^ NaCl aggregating agent were used for SERS, and the collection time is 30 s

Examples of the SERS spectra for the optimised SERS conditions for each of the analytes are shown in Fig. 4, and Table 1 provides the tentative band assignments for caffeine, theobromine and paraxanthine.

**Table 1:** Tentative band assignment for SERS spectra of caffeine, theobromine and paraxanthine

	Raman shift (cm^−1^)	Assignment
Caffeine and paraxanthine	509	N–C–C deformation ^(1)^
	650	O=C–N deformation ^(1)^
	693	C=O deformation ^(1)^
	1006	N–CH_3_ asymmetric stretch ^(1)^
	1247	C–N stretch ^(1)^
	1450	*δ*(CH_3_) ^(2)^
	1672	Unassigned
	2953	*ν*(CH_3_) ^(2)^
Theobromine	637	*δ*(pyrimidine, imidazole ring) + *δ*(CH_3_) + *ρ*(CH_3_) ^(3)^
	1228	*δ*(CH–N) + *ρ*(CH_3_) ^(3)^
	1303	*ν*(C–N) + *ρ*(CH_3_) ^(2)^
	1353	*δ*(CH–N) + *ρ*(CH_3_) ^(3)^
	1413	*δ*(CH_3_) ^(3)^
	1603	*ν*(C=C) + *ν*(C–N) + *δ*(CH_3_) ^(3)^
	–	
	2953	*ν*(CH_3_) ^(2)^

### SERS analysis of tertiary mixtures

Now that we have established the optimal SERS conditions to be used, prior to tertiary mixture analysis, we first conducted serial dilutions of each of the analytes individually to calculate the working concentration range. Each analyte was diluted from 10^−2^ to 10^−7^ mol/dm^3^, and five repeats for each measurement were made. Peak areas for the bands at 693 cm^−1^ from C=O deformation for caffeine and paraxanthine and 1303 cm^−1^ from *ν*(C–N) + *ρ*(CH_3_) for theobromine were calculated. Plots against concentrations of each of the analytes (ESM Fig. S5) showed reasonable linearity for all analytes between 10^−5^ and 10^−7^ mol/dm^3^ for theobromine and paraxanthine and between 10^−4^ and 10^−6^ mol/dm^3^ for caffeine, respectively. This higher working range for caffeine is likely to be due to the slower dynamics of association (ESM Fig. S4).

Based on the above, we decided to make three-way mixtures of the three analytes from 10^−5^ to 10^−7^ mol/dm^3^. The number of molecules in the reaction was consistently kept to 10^−5^ mol/dm^3^ (10 μmol/L), and the preparation of the 66 mixtures (at ‘10 %’ range intervals) is illustrated in ESM Table S1. All mixtures were prepared in triplicate. All 198 samples were analysed over a period of 5 days using the following optimised protocol:

- The reaction pH was 8.
- The analyte-silver citrate colloid association time was 10 min.
- 1.0 mol/dm^3^ NaCl aggregating agent was used and vortexed for 10 s.
- SERS spectra were collected immediately at 633 nm with ∼3 mW power on the sample.

### ANN mixture modelling

Prior to any neural network modelling, the SERS data were baseline corrected using an ALS smoothing algorithm technique after which SNV was performed [17].

As neural networks are a supervised learning method, it is important that they are not over trained. That is to say, they have learnt the training data too perfectly and are not able to generalise to an independent test set. In order to assess the ability of ANNs to predict the concentrations of the three analytes, we performed bootstrap validation and all the data generated here are the results from 1000 test sets.

**Fig. 5:**
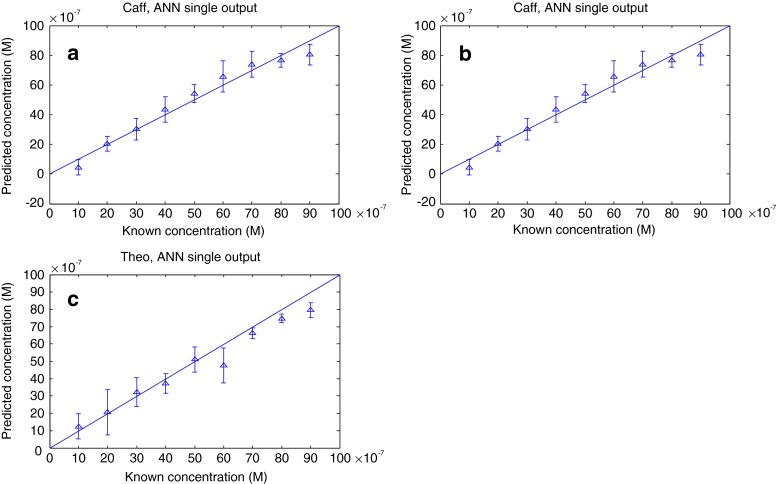
Artificial neural network predictions for three individual models trained to quantify the levels of caffeine (**a**), theobromine (**b**) and paraxanthine (**c**). *Points* are the averages from the 1000 test set bootstraps, and *error bars* show the standard deviations

We initially calibrated three ANNs with the topology 1024-20-1 where the input was the total SERS spectra, and we used 20 nodes in the hidden layer and a single output node. The results for the 1000 bootstrap test sets are shown in Fig. 5. It is clear that the predicted concentrations versus the actual concentrations are very close to the expected *y* = *x* line. Indeed, the ANNs’ ability to predict each of the three analytes are very good and the statistics from these models (Table 2) shows that the error in the predictions for theobromine (RMSEP 9 × 10^−7^ mol/dm^3^) and paraxanthine (1 × 10^−6^ mol/dm^3^) is lower than that for caffeine (2 × 10^−6^ mol/dm^3^) and that the correlation coefficient for the test sets (*Q*^2^) are 0.9 for theobromine and paraxanthine, and this drops to 0.7 for caffeine. This is perhaps to be expected, given that caffeine associates with the surface slower than either theobromine or paraxanthine.

**Table 2:** Artificial neural network predictions for caffeine, theobromine and paraxanthine. ANNs were constructed with either a single output node (one model for each of the analytes) or using three output nodes in a single ANN

	Caffeine	Theobromine	Paraxanthine
Single output node in a 1024-20-1 topology			
*R* ^2^	0.9999	0.9939	0.9884
*Q* ^2^	0.7067	0.8958	0.8953
RMSEP (mol/dm^3^)	1.6832 × 10^−6^	8.8402 × 10^−7^	9.6140 × 10^−7^
Three output nodes in a 1024-20-3 topology			
*R* ^2^	0.9999	0.9999	0.9999
*Q* ^2^	0.8093	0.8115	0.8785
RMSEP (mol/dm^3^)	1.3222 × 10^−6^	1.3145 × 10^−6^	1.0553 × 10^−6^

We also conducted additional ANN analyses where the ANN was calibrated to predict the three analytes simultaneously. The network architecture was 1024-20-3 where the three output nodes were used for caffeine, paraxanthine and theobromine. It was clear from the statistics on the 1000 bootstraps (Table 2) that the predictions were not as accurate as the three individual models, a phenomenon that is routinely seen with mixture modelling [17, 30].

## Concluding remarks

Measuring drugs and their metabolites are important as they can be used to establish long-term abuse of illicit materials. This is normally achieved using targeted LC-MS and therefore involves large expensive equipment and is time consuming and labour intensive. For remote testing, there is therefore the need for small portable analytical techniques. In order to investigate this, we chose the model drug caffeine with its two major metabolomics theobromine and paraxanthine and selected Raman spectroscopy as the analytical method as it is reproducible, robust and portable. However, the signal from the normal Raman scattering process is too weak for this to be practical. Therefore, we developed colloid-based SERS for the quantitative analysis of these three analytes in tertiary mixtures without recourse to prior chromatographic separation. SERS spectra were obtained using a portable Raman probe (DeltaNu instrumentation), the multivariate data generated were analysed with ANNs, and this allowed for the simultaneous quantification of the drug caffeine and its xenometabolites theobromine and paraxanthine. The predictions generated between 10^−5^ and 10^−7^ mol/dm^3^ were excellent for all the three analytes which are easily within the blood and urine concentrations for caffeine and its major metabolites (maximum of ca. 0.5–10 μM 1.2 h after coffee consumption [19]). Future studies will be conducted to extend this to testing from complex biological matrices, including blood and urine, where we expect that we shall have to use selective solvent extraction to recover these pyridine-based chemical species.

## Electronic supplementary material

ESM 1 (PDF 164 kb)
