# Supplementary material for: Simultaneous multiplexed quantification of caffeine and its major metabolites theobromine and paraxanthine using surface-enhanced Raman scattering
Source: Anal Bioanal Chem. 2015 Sep 7;407(27):8253–61. doi: 10.1007/s00216-015-9004-8 (PMC4604501; doi:10.1007/s00216-015-9004-8)
Supplement: Supplementary file 1 — (PDF 164 kb) [file 216_2015_9004_MOESM1_ESM.pdf]

Analytical and Bioanalytical Chemistry

Electronic Supplementary Material

Simultaneous multiplexed quantification of caffeine and its major metabolites theobromine and paraxanthine using surface-enhanced Raman scattering

Omar Alharbi, Yun Xu, Royston Goodacre

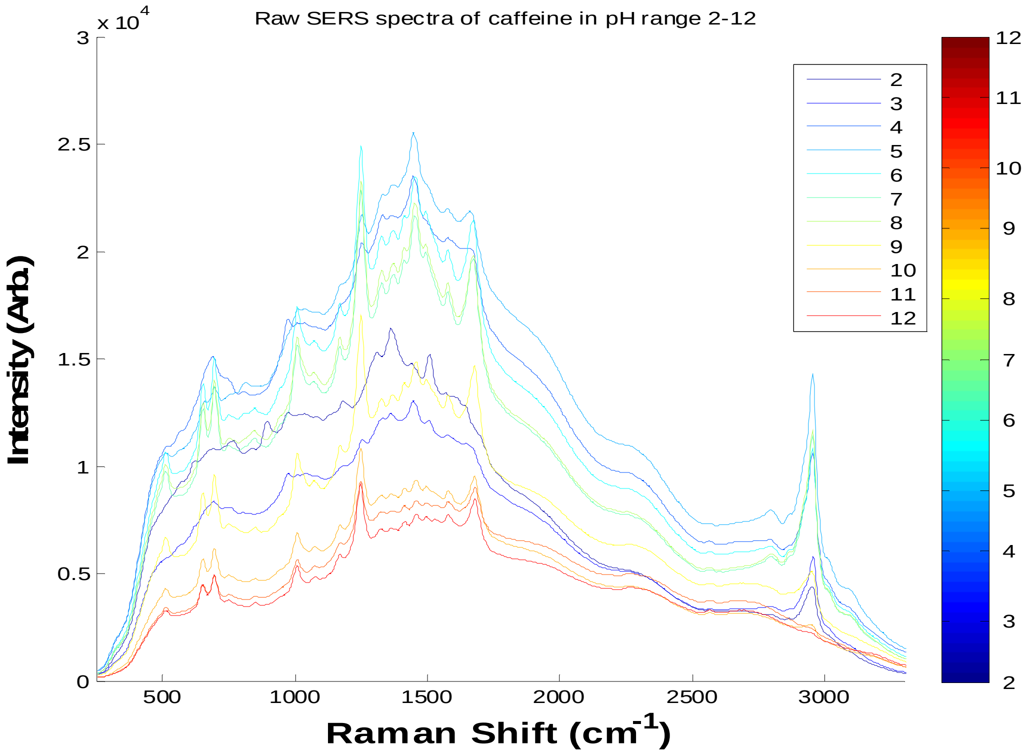

Fig. S1 Raw SERS spectra of caffeine in pH range 2-12

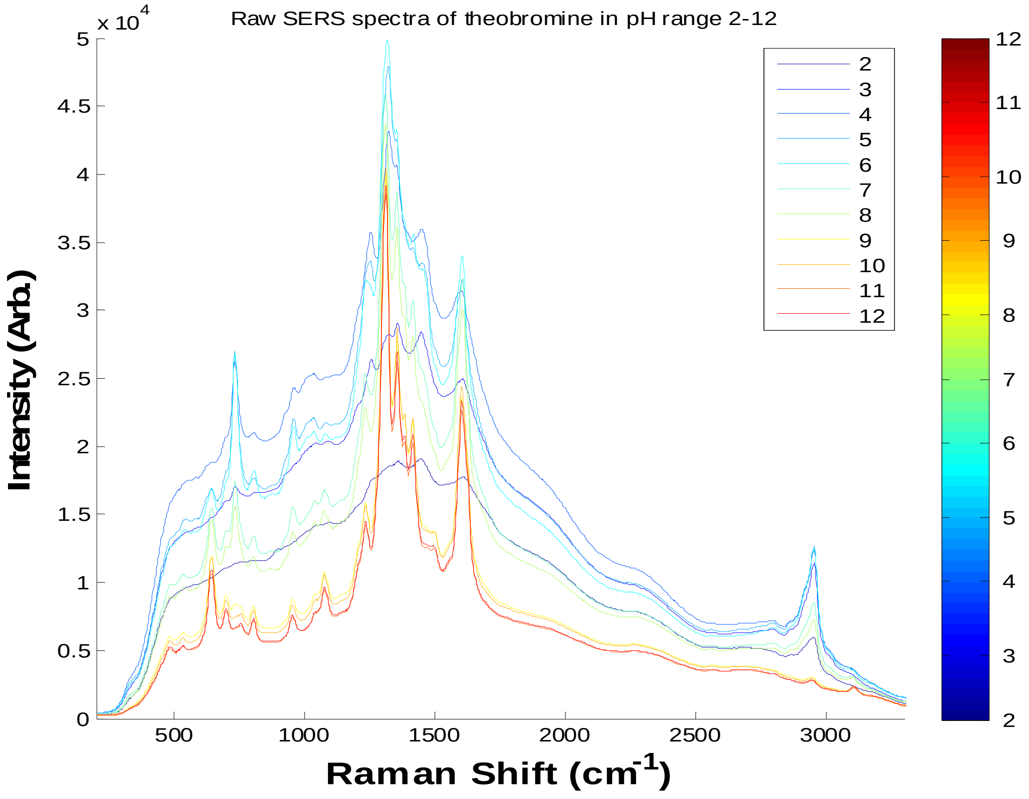

Fig. S2 Raw SERS spectra of theobromine in pH rang 2-12

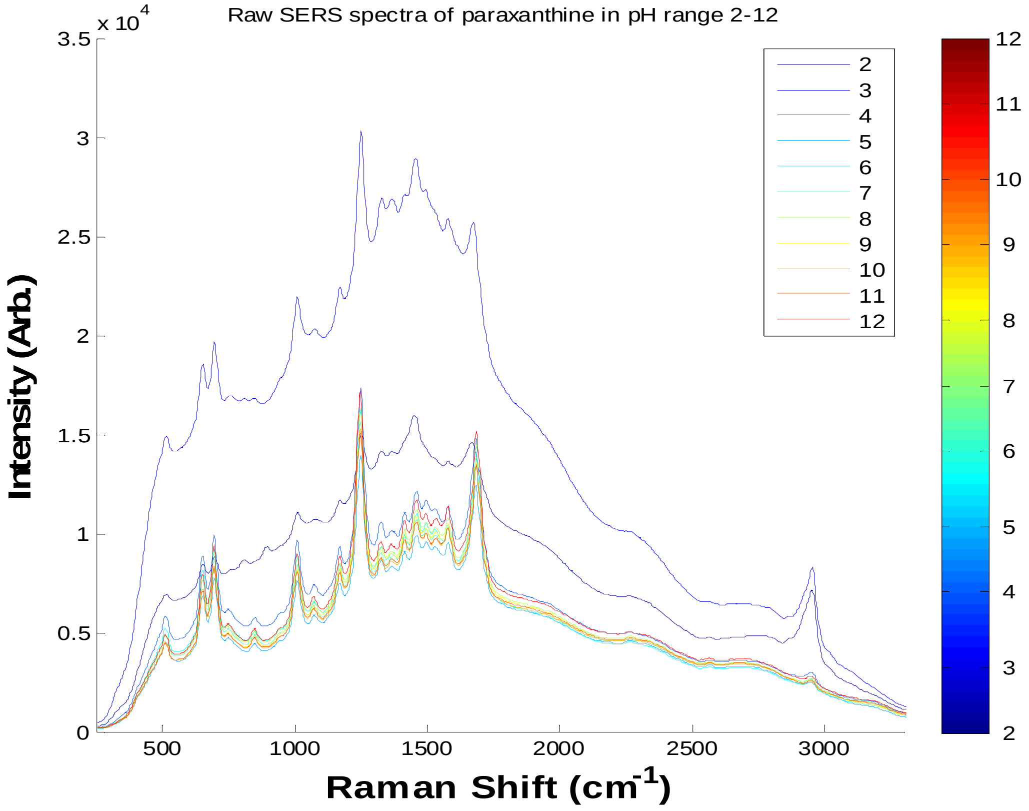

Fig. S3 Raw SERS spectra of paraxanthine in pH range 2-12

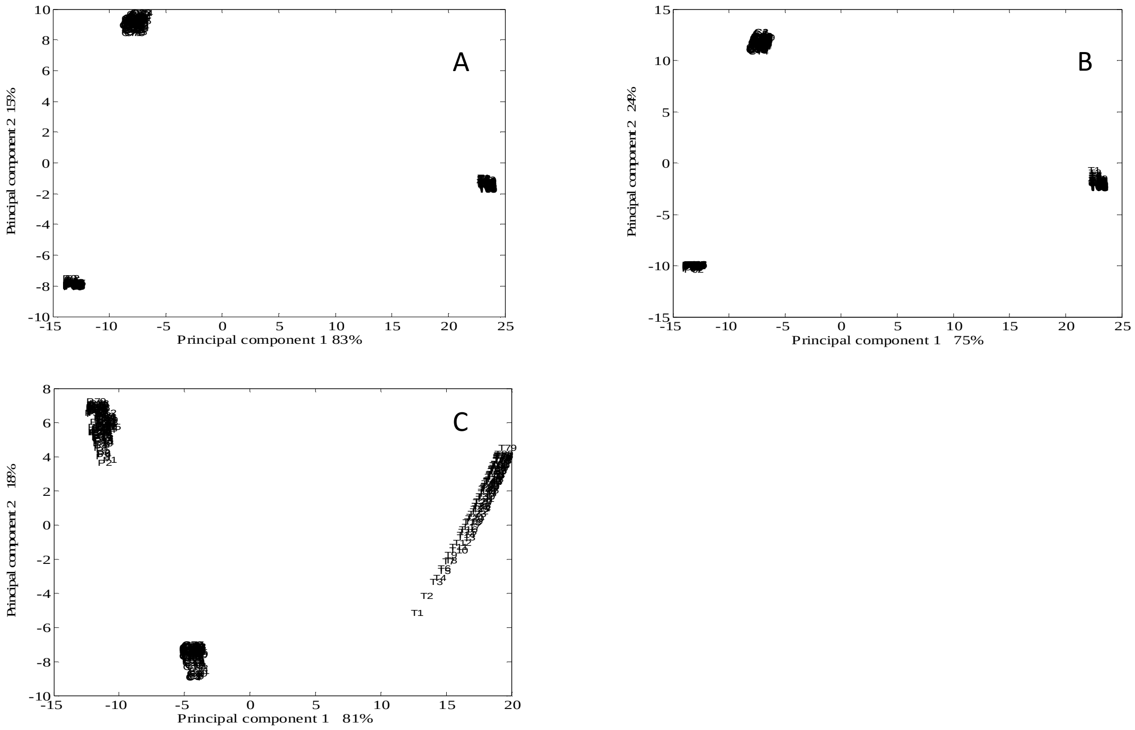

Fig. S4 PCA scores plots of PC1 *versus* PC2 for Batches 1 (A), 2 (B) and 3 (C) of silver citrate colloid. The symbols used refer to Xn, where X is either C (caffeine), (P) paraxanthine or T (theobromine) and the n refers to the order in which the spectral data have been collected. The percentage total explained variance for each of the PCs is also shown in the axes legends

For Figures S5-S7 limits of detection (LoD) were calculated using procedures detailed in Mabbott, S., Alharbi, O., Groves, K. & Goodacre, R. (2015) Application of surface enhanced Raman scattering to the solution based detection of a popular legal high, 5,6-methylenedioxy-2-aminoindane (MDAI). *Analyst* 140, 4399-4406.

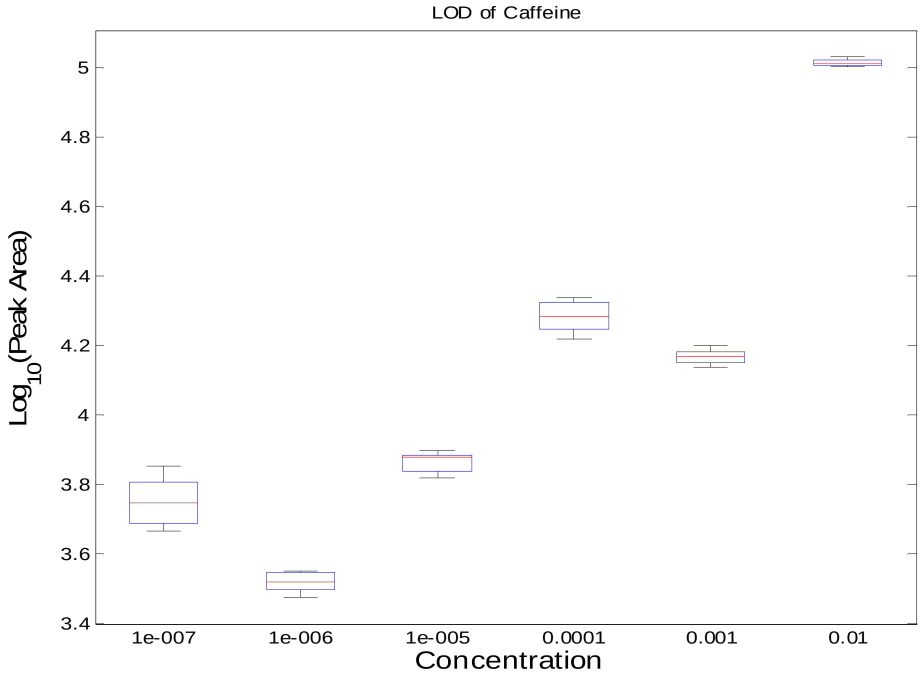

Fig. S5 Plot of Peak area for 693 cm^{-1} against [caffeine]; this was used to establish the limit of detection and the practical useful linear concentration range

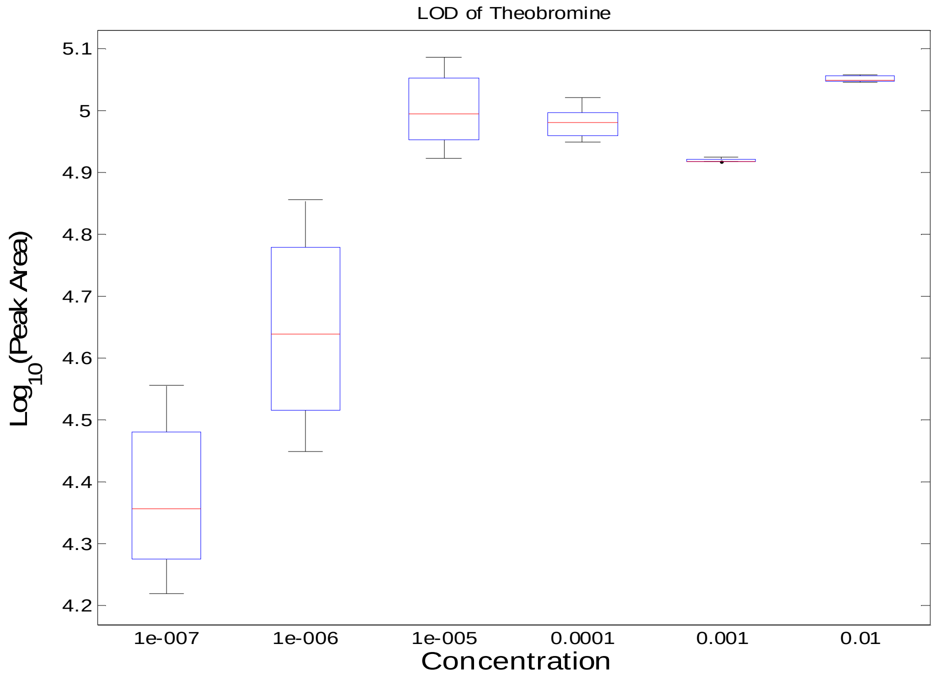

Fig. S6 Plot of Peak area for 1303 cm^{-1} against [theobromine]; this was used to establish the limit of detection and the practical useful linear concentration range

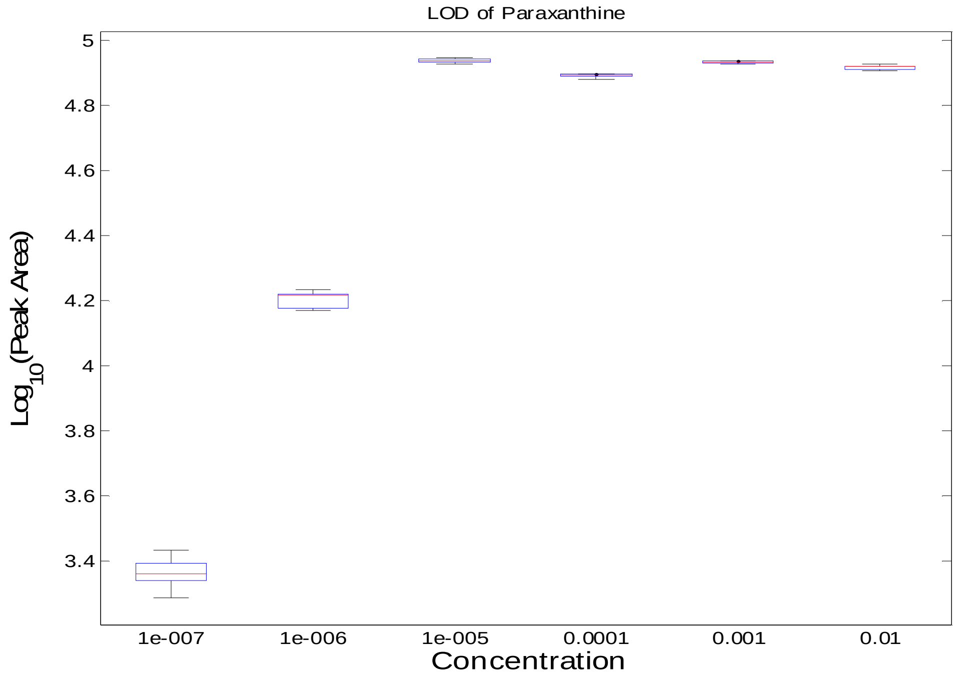

Fig. S7 Plot of Peak area for 693 cm^{-1} against [paraxanthine]; this was used to establish the limit of detection and the practical useful linear concentration range

Table S1 Examples of some of the tertiary mixtures and how they were generated from 3 stock solutions of caffeine, theobromine and paraxathine. For these experiments the total molarity in each tube was constant at 1.10^{-5} mol/m^3 (10 µmol/L)

Caff (%)	Theo (%)	Para (%)	Caff (µL)	Theo (µL)	Para (µL)	Sample number		
0	0	100	0	0	200	1	2	3
0	10	90	0	20	180	4	5	6
0	20	80	0	40	160	7	8	9
0	30	70	0	60	140	10	11	12
0	40	60	0	80	120	13	14	15
0	50	50	0	100	100	16	17	18
0	60	40	0	120	80	19	20	21
0	70	30	0	140	60	22	23	24
0	80	20	0	160	40	25	26	27
0	90	10	0	180	20	28	29	30
0	100	0	0	200	0	31	32	33
10	0	90	20	0	180	34	35	36
10	10	80	20	20	160	37	38	39
10	20	70	20	40	140	40	41	42
…	…	…	…	…	…	*n*	*n*	*n*
90	10	0	180	20	0	193	194	195
100	0	0	200	0	0	196	197	198
